# Left ventricular strain during exercise stress: a CMR myocardial feature tracking study

**DOI:** 10.1186/1532-429X-18-S1-P39

**Published:** 2016-01-27

**Authors:** Vera-Christine Stahnke, Johannes T Kowallick, Jan M Sohns, Michael Steinmetz, Antonia Zapf, Joachim Lotz, Andreas Schuster, Christina Unterberg-Buchwald

**Affiliations:** 1 Institute of Interventional and Diagnostic Radiology, University Clinic Goettingen, Goettingen, Germany; 2 Dept. of Pediatric Cardiology, University Clinic Goettingen, Goettingen, Germany; 3 grid.7450.60000000123644210Institute of Medical Statistics, University Goettingen, Goettingen, Germany; 4 Dept. of Cardiology and Pneumology, University Clinic Goettingen, Goettingen, Germany

## Background

Abnormalities in left ventricular (LV) strain as detected during pharmacological or physiological exercise stress have been demonstrated to be earlier and more sensitive markers of contractile dysfunction than global ejection fraction. Recent developments allow for in-scanner exercise using MR-compatible ergometers. Therefore, the objective of this study was to analyze LV strain with cardiovascular magnetic resonance myocardial feature tracking (CMR-FT) in volunteers during exercise using an in-scanner ergometer.

## Methods

15 healthy volunteers were enrolled for supine cycle ergometry on the CMR scanner table using a MR-compatible ergometer (Lode, The Netherlands). Imaging was performed at 3T (Siemens Skyra). Long-axis 2- and 4-chamber steady state free precession (SSFP) cine images as well as short-axis stacks were acquired at rest and after 3 minutes of cycling at 50W and 100W during a 30 sec break to minimize motion artifacts. CMR-FT (TomTec Imaging Systems, Germany) was performed in 2- and 4-chamber views to quantify left ventricular global longitudinal strain (EII), and in all short-axis slices to quantify global radial strain (Err) and global circumferential strain (Ecc). Furthermore, LV volumes and ejection fraction (EF) were analyzed from the whole short-axis stack (Qmass, Medis, The Netherlands).

## Results

Results are displayed in table 1. Heart rate continuously increased with increasing exercise levels. EF increased with moderate exercise and remained stable at higher exercise levels. CMR-FT was successfully performed in all subjects at rest. After 50W and 100W exercise, cine SSPF images of two and four volunteers had to be excluded due to considerable breathing artifacts, respectively. Values for Ecc, Err and Ell increased significantly from rest to 50W. No additional increase in strain was observed between 50W and 100W.

**Table 1:** Left ventricular volumes, function and strain in comparison between rest and exercise with 50 and 100 W, respectively. Values are given as mean ± standard deviation

	Rest	50 W	100W	50 W vs rest	100 W vs rest	100W vs 50W
				p-values		
**HF [beats/min]**	59.6 ± 12.4	93.8 ± 14.8	116.8 ± 12.3	**<0.001**	**< 0.001**	**< 0.001**
**EDVI [ml/m** ^**2**^ **]**	86.3 ± 12.2	90.7 ± 8.2	80.7 ± 13.0	0.177	0.201	**0.004**
**ESVI [ml/m** ^**2**^ **]**	29.1 ± 5.3	23.9 ± 6.0	21.7 ± 6.8	**<0.001**	**<0.001**	<0.07
**SVI [ml/m** ^**2**^ **]**	57.5 ± 10.7	66.8 ± 5.0	59.8 ± 13.1	**0.01**	0.568	0.07
**EF [%]**	66.4 ± 5.6	73.9 ± 4.6	72.9 ± 9.0	**<0.001**	**0.025**	0.501
**Ecc [%]**	-20.4 ± 2.7	-26.4 ± 2.8	-27.7 ± 2.7	**<0.001**	**<0.001**	0.146
**Ell [%]**	-18.9 ± 2.9	-26.5 ± 4.5	-27.9 ± 4.4	**< 0.001**	**< 0.001**	0.311
**Err [%]**	26.8 ± 4.9	30.3 ± 5.2	33.5 ± 9.4	**0.031**	**0.034**	0.12

EDVI, enddiastolic volume index; ESVI, endsystolic volume index; SVI, stroke volume index; EF, ejection fraction; HR, heart rate; Ecc, global circumferential strain; Err, global radial strain; Ell, global longitudinal strain. Bold p values indicate a significance level < 0.05 as determined by Students's paired t-test.

## Conclusions

CMR-FT derived quantification of LV strain is feasible during dynamic exercise using a supine MR-compatible ergometer. It provides a potential technique for assessing radial, circumferential and longitudinal LV strain in different patient groups during physiological exercise.

